# Causal relationship between genetically predicted Enzyme and Tinnitus risk: a two-sample bi-directional mendelian randomization study

**DOI:** 10.1016/j.bjorl.2026.101804

**Published:** 2026-03-26

**Authors:** Peng Liu, Xinmiao Xue, Zhixin Zhang, Lijun Zhang, Yu Peng, Qi Song, Fangyuan Wang

**Affiliations:** a Bethune International Peace Hospital, Department of Otolaryngology Head and Neck Surgery, Shijiazhuang, Hebei Province, P.R. China; b Chinese PLA General Hospital, Institute of Otolaryngology, Department of Otolaryngology, Head and Neck Surgery, Beijing, P.R. China

**Keywords:** Enzyme, Tinnitus, Mendelian randomization

## Abstract

**Objectives:**

Observational studies have reported an association between enzymes and tinnitus. However, the exact nature and strength of the causal relationship between enzymes and tinnitus remain unclear.

**Methods:**

In this study, we utilized two-sample bi-directional mendelian randomization using publicly available GWAS summary statistics to investigate the causal relationship between enzymes and the risk of tinnitus. Causal relationship was estimated using the inverse variance weighted method. In additional, the validity of the causal relationship was assessed by conducting sensitivity tests.

**Results:**

After accounting for heterogeneity and horizontal pleiotropy, we found suggestive evidence supporting the causality of nine enzymes on tinnitus including 3-hydroxy-3-methylglutaryl-coenzyme A reductase, Alanine-tRNA ligase cytoplasmic, Fatty-acid amide hydrolase 2, E3 ubiquitin-protein ligase DTX1, E3 ubiquitin-protein ligase ZNRF3, Glutathione S-transferase A3, Liver enzyme levels (alanine transaminase), Platelet-activating factor acetylhydrolase IB subunit beta, Probable inactive ribonuclease-like protein 13. Furthermore, evidence of causal effects of tinnitus on six types of enzymes including Serine protease 27 levels, Phospholipase D3 measurement, Inactive peptidyl-prolyl cis-trans isomerase FKBP6, Probable E3 ubiquitin-protein ligase MID2, Ubiquitin-like modifier-activating enzyme ATG7, Aldo-keto reductase family 1 member C1 was also detected.

**Conclusions:**

The present study suggests a causative effect of specific types of enzymes on tinnitus, as well as a reverse causality between them. These will provide potential targets for the treatment of tinnitus and enrich strategies to alleviate tinnitus-related comorbidities.

**Level of evidence:**

I.

## Highlights

• The causal relationship between enzymes and tinnitus remain unclear.
• 5 proteases have protective effect on tinnitus and 4 proteases promote tinnitus.
• Tinnitus reduce the content of 2 proteases and increase the content of 4 proteases.
• A foundation for finding effective treatment targets for tinnitus was laid.

## Introduction

Tinnitus, a prevalent condition, is characterized by the perception of sounds by patients in the absence of external stimuli.^1^ Previous surveys conducted in the United States and Western Europe have reported an incidence rate of 10%‒15% among normal adults.^2^ Among the above individuals, 2.8% experience mild tinnitus symptoms, while 1.6% have their daily lives severely affected by the disease. Studies depicted that a strong association exists between tinnitus and hearing loss, however, not all patients with tinnitus combined with hearing impairment.^3^ The development of tinnitus is an intricate process that remains poorly understood. Due to its elusive pathogenesis, it currently lack of effective treatment options, and traditional interventions including drugs and cognitive behavioral therapy have shown limited therapeutic efficacy for tinnitus.^4^ Therefore, identifying and illuminating the specific therapeutic targets for tinnitus is of utmost importance.

As crucial regulators of physiological reactions, enzymes play a vital role in catalyzing substance synthesis and promoting metabolism.^5^^,^^6^ Numerous studies have reported that there is a close association between enzymes and various diseases, such as digestive diseases, respiratory diseases, metabolic diseases, and nervous system diseases.^7^, ^8^, ^9^, ^10^ The relationship between enzymes and the development of tinnitus has also been investigated.^11^ Study based on the serum oxide content, discovered that the serum prolinase content was higher in tinnitus patients compared to normal individuals.^12^ In addition, a strong association between ACE I/D and α-increasing protein (ADD1) G460W gene polymorphisms exists in patients with chronic tinnitus.^13^ Furthermore, tinnitus patients exhibited reduced levels of salivary alpha-amylase due to chronic stress.^14^ Additionally, KANG et al. found a significant correlation between acetyltransferase and the severity of tinnitus.^15^ However, when antioxidants were applied to clinical patients, no significant changes in oxidative stress markers associated with tinnitus were observed.^16^ Therefore, the precise causal relationship between enzymes and tinnitus remains controversial. Investigating the causal relationship between enzymes and tinnitus is crucial for effectively predicting and treating tinnitus.

Because the relationship between exposure and outcome of diseases is affected by many factors, including bias factors, confounders and reverse causation factors, traditional observational epidemiological studies have certain drawbacks. At the same time, there are also the disadvantages of time consuming, laborious data acquisition and long observation period in observational epidemiological studies. As a new epidemiological method, Mendelian Randomization (MR) uses Instrumental Variables (IVs) that have a strong relationship with exposure factors to explore the causal relationship between exposure and outcome of disease. It can effectively avoid the influence of confounding factors and reverse causality, and the explanation based on genetic variation is also superior to the explanation based on outcome.^17^

In this study, the Genome-Wide Association Studies (GWAS) dataset of enzymes and tinnitus was utilized for two-sample MR analysis, aiming to explore whether there is a causal relationship between them. These findings provide a basis for researchers to search for specific biomarkers related with tinnitus, and also contribute to the prevention and treatment of tinnitus.

## Methods

### Study design

**Fig. 1:**
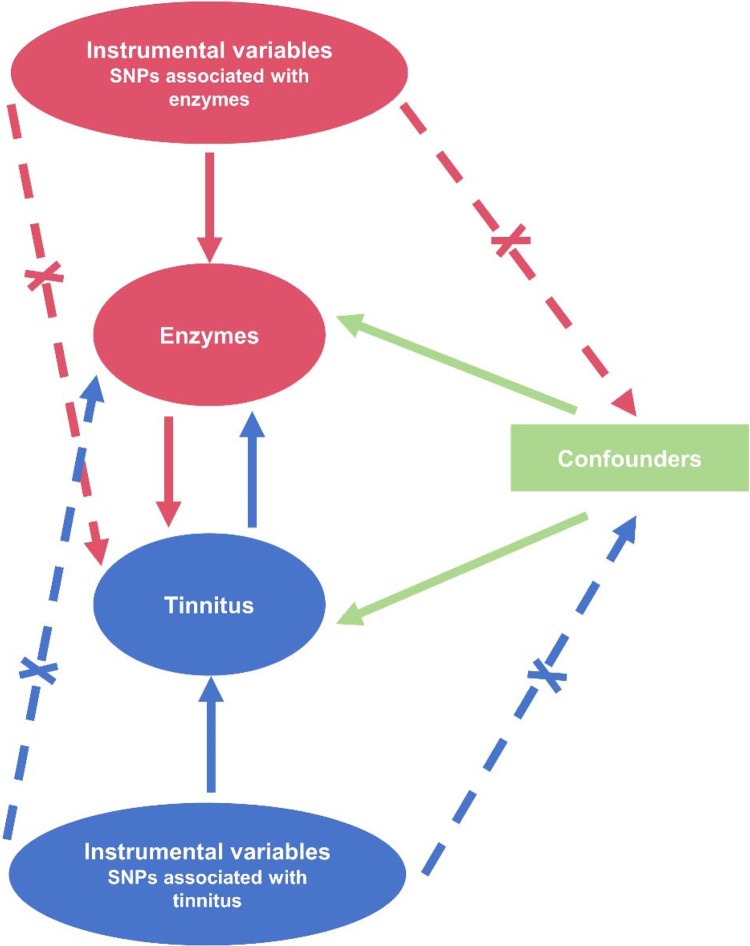
Plot of the study design for bidirectional mendelian randomization between enzymes and tinnitus. The red solid lines depict the association between instrumental variables (SNPs) and exposure, as well as the association between exposure and outcome. The blue solid lines represent the association of reverse causality. The dashed lines with a cross symbol indicate that the association satisfies the two fundamental assumptions of mendelian randomization: (i) The genetic variants (SNPs) are independent of confounders between exposure and outcomes; (ii) The genetic variants only influence the outcome through exposure.

A two-sample MR method was utilized to investigate the causal relationship between 233 enzymes and the risk of tinnitus. The concrete study workflow is shown in Fig. 1. Firstly, association data for the 233 enzymes (exposure variable) were obtained from GWAS database. The Single Nucleotide Polymorphisms (SNPs) associated with these enzymes were identified. Subsequently, another GWAS database was utilized to obtain the associated data for tinnitus (outcome variable) and identify the presence of related SNPs. Finally, qualified SNPs were selected, and the causal association between the enzymes and the risk of tinnitus was evaluated using various statistical methods. Additionally, the causal relationship between tinnitus (exposure variable) and the 233 enzymes (outcome variable) was also examined.

### Data source

The summary data for tinnitus-related information was obtained from the IEU open GWAS database (HTTPS / / GWAS. Mrcieu. Ac. UK/datasets). The total sample size was 5070, with a total of 9806006 SNPs. The enzyme-related data were compiled from the GWAS catalog, which includes proteases, hydrolases, oxidase enzymes, ligases, lipases, and enzymes necessary for various important physiological processes. Moreover, the dataset was exclusively derived from the European cohort and did not overlap with other demographics. Further details regarding this dataset can be found in the supplementary material 1. The data were anonymized and publicly available, thus consent from the participants were not required.

### Selection of SNPs

For enzymes as exposure, the selection and identification of SNPs with genome-wide significance is based on a threshold of p < 5 × 10^−8^. This ensures that only SNPs with a high level of statistical significance are considered. In addition, a Linkage Disequilibrium (LD) parameter of  *r*^2^ < 0.001 and a genetic distance of 10 MB are used to ensure the independence of instrumental variables. The SNPs with the smallest p-value are selected to ensure the validity of the instrumental variables. The data from two databases are then extracted and consolidated, ensuring that the effect values of exposure and outcome correspond to the same effect allele. The strength of the instrumental variables is evaluated using the F statistic, F = (β/se) 2, where β represents the allele effect value and se is the standard error. A threshold of F > 10 is used to identify instrumental variables without weak instruments. For tinnitus as exposure, the screening criteria are the same as mentioned above, except that a less stringent threshold of p < 5 × 10^−7^ is used.

### Statistical analysis

In this study, the relationship between enzymes and tinnitus is analyzed using the Inverse Variance Weighted analysis (IVW) method. Additionally, MR-Egger, Weighted Median (WM), and Penalized Weighted Median (PWM) methods are employed to further validate the results obtained from IVW. The statistical analysis is performed using the Two Sample MR Software packages, with a significance level of α = 0.05. Sensitivity analysis includes heterogeneity testing, horizontal pleiotropy testing, and individual elimination testing. Heterogeneity testing is primarily used to detect differences among instrumental variables and is conducted using both the IVW and MR-Egger methods. The presence of horizontal pleiotropy is assessed through the intercept term of the MR-Egger method. A p-value greater than 0.05 indicates no evidence of horizontal pleiotropy. The one-by-one elimination test involves calculating the combined effect of the remaining SNPs after sequentially removing one SNP at a time.

## Results

### Causal effect of enzyme on tinnitus

**Fig. 2:**
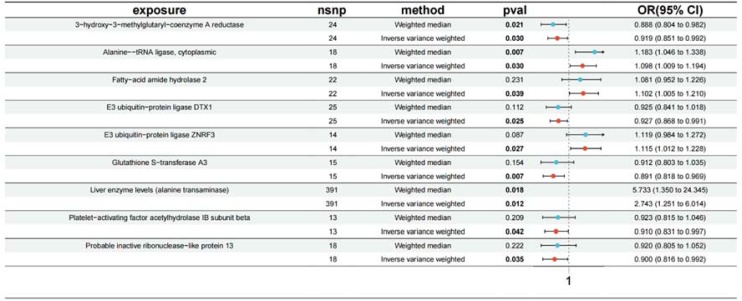
Forest plot presenting the causal estimates of enzymes on tinnitus.

All SNPS were strong instrumental variables, and no weak instrumental variables biased the results. MR Analysis (IVW) results show that the following enzyme including 3-hydroxy-3-methylglutaryl-coenzyme A reductase (OR = 0.919; 95% CI [0.851, 0.992], p = 0.030), Alanine-tRNA ligase, cytoplasmic (OR = 1.098; 95% CI [1.009, 1.194], p = 0.030), Fatty-acid amide hydrolase 2 (OR = 1.102; 95% CI [1.005, 1.210], p = 0.039), E3 ubiquitin-protein ligase DTX1 (OR = 0.927; 95% CI [0.868, 0.991], p = 0.025), E3 ubiquitin-protein ligase ZNRF3 (OR = 1.115; 95% CI [1.012, 1.228], p = 0.027), Glutathione S-transferase A3 (OR = 0.891; 95% CI [0.818, 0.969], p = 0.007), Liver enzyme levels (alanine transaminase) (OR = 2.743; 95% CI [1.051, 6.014], p = 0.012), Platelet-activating factor acetylhydrolase IB subunit beta (OR = 0.091; 95% CI [0.831, 0.997], p = 0.042), Probable inactive ribonuclease-like protein-13 (OR = 0.900; 95% CI [0.816, 0.992], p = 0.035) have causal effect on tinnitus shown in Fig. 2.

### Causal effect of tinnitus on enzyme

**Fig. 3:**
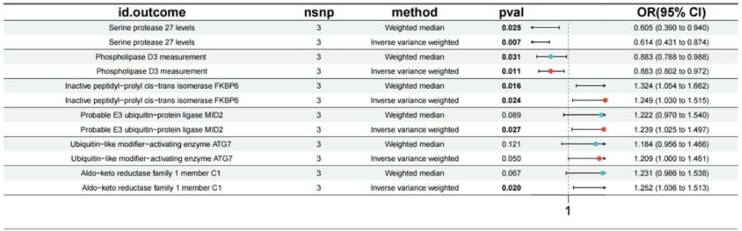
Forest plot presenting the causal estimates of tinnitus on enzymes.

With respect to the effect of tinnitus on enzyme, Serine protease 27 levels (OR = 0.614; 95% CI [0.431, 0.874], p = 0.007), Phospholipase D3 measurement [OR = 0.883; 95% CI [0.802, 0.972], p = 0.011), Inactive peptidyl-prolyl cis-trans isomerase FKBP6 (OR = 1.249; 95% CI [1.030, 1.515], p = 0.024), Probable E3 ubiquitin-protein ligase MID2 (OR = 1.239; 95% CI [1.025, 1.497], p = 0.027), Ubiquitin-like modifier-activating enzyme ATG7 (OR = 1.209; 95% CI [1.000, 1.461], p = 0.050), Aldo-keto reductase family 1 member C1 (OR = 1.252; 95% CI [1.036, 1.513], p = 0.020) were found as shown in Fig. 3.

## Discussion

Currently, there is a lack of effective treatments for tinnitus especially for the chronic subtypes when lasting for more than 6-months. Therefore, it is crucial to identify suitable therapeutic targets and biomarkers to effectively prevent and treat this disease. Enzymes, acting as catalysts within the body, are involved in both normal and abnormal physiological functions, making their alteration closely associated with the occurrence and progression of diseases. In this study, a bi-directional MR method was employed, with enzymes as the exposure factor and tinnitus as the outcome factor, to investigate the impact of enzymes on tinnitus as well as the possibility of reverse causality. Through the analysis of SNPs, it was discovered that 5 enzymes had a protective effect on tinnitus, while 4 enzymes had a deteriorative effect. Additionally, the study revealed that tinnitus impacts 6 enzymes, with 2 of these enzymes exhibiting negative regulation in the presence of tinnitus, contrasted by the remainder which are subject to positive regulation.

Previous studies have confirmed the close relationship between HMG-CoA reductase and the synthesis of CoQ10.^18^ CoQ10 plays a crucial role in regulating the expression of multiple genes involved in mitochondrial metabolism and inflammation.^19^ Upregulation of HMG-CoA reductase can enhance mitochondrial function, reduce mitochondrial degradation, and decrease reactive oxygen species and inflammatory reactions, all of which are closely associated with the occurrence of tinnitus.^20^ Therefore, HMG-CoA reductase can serve as a protective factor against tinnitus.

Alanine-tRNA ligase primarily participates in the translation and metabolism of RNA and proteins.^21^ Consequently, an increase in alanine-tRNA ligase may lead to excessive synthesis of certain proteins in the central nervous system, resulting in cellular excitotoxicity and subsequently causing tinnitus.^22^ Fatty-Acid Amide Hydrolase 2 (FAAH) is intimately involved in the degradation of endocannabinoids in vivo.^23^ Previous studies have reported that the endocannabinoid system can alleviate tinnitus. FAAH may induce tinnitus by influencing the levels of endocannabinoid components in vivo.^24^

Deltex1 (DTX1) is closely associated with the Notch1 signaling pathway, and it has been discovered that DTX1 can regulate ERB2 transcription and glial cell differentiation by modulating the Notch1 signaling pathway.^25^^,^^26^ Previous studies have also reported the impact of glial cells on tinnitus which suggests that DTX1 can regulate the occurrence of tinnitus.^27^

Zinc and Ring Finger 3 (ZNRF3), another member of the ubiquitination family, can inhibit the WNT signaling pathway by promoting the flipping of LDL-associated proteins.^28^ The WNT signaling pathway is down-regulated in neurodegenerative diseases such as Alzheimer's Disease and Parkinson's disease.^29^ Since tinnitus is considered a subtype of neurodegenerative diseases that share potential genes,^30^^,^^31^ ZNRF3 can inhibit the WNT signaling pathway to promote tinnitus.

Glutathione plays a crucial role in fighting oxidative stress, which is closely related to the development of tinnitus.^32^^,^^33^ Glutathione acts as a protective factor for this auditory hallucination may through the above mechanism.

Alanine transferase, on the other hand, helps maintain the level of glutamate in the body during pathological processes like ischemia/hypoxia.^34^ However, despite its potential neuroprotective effect,^35^ the accumulation of glutamate in the auditory cortex can worsen tinnitus.

The Platelet-activating factor acetylhydrolase IB subunit beta complex, commonly referred to as the Lissencephaly-1 complex, serves as a pivotal lipid second messenger. It facilitates a diverse range of biological processes and is particularly enriched in the mammalian central nervous system. Within this system, Platelet-activating factor functions as a synaptic messenger, a transcription inducer, and is implicated in long-term potentiation ‒ a fundamental synaptic mechanism underlying memory formation.^36^ Recent research has elucidated that pyramidal neurons expressing Lissencephaly-1 are crucial for the preservation of parvalbumin + inhibitory interneurons and a reduction in the above neurons has been significantly associated with the onset of tinnitus.^37^^,^^38^

Probable inactive ribonuclease-like protein-13 is known to play a role in DNA repair mechanisms. In instances of DNA damage, probable inactive ribonuclease-like protein-13 contributes to the repair process by eliminating damaged RNA at the site of lesion.^39^ The protective role of probable inactive ribonuclease-like protein-13 might be exerted through its function in DNA repair, as individuals experiencing hearing loss and tinnitus have been observed to have a higher mean level of DNA damage.^40^

We conducted a similar investigation on the impact of tinnitus on proteases in the body. One of the enzymes we focused on is phospholipase C, which plays a crucial role in modifying phospholipids and regulating cell growth, differentiation, and endocytosis.^41^^,^^42^ Previous studies have demonstrated a decrease in the expression of this enzyme in the brain tissue of Alzheimer's patients.^43^ Therefore, the reduced levels of phospholipase C in tinnitus patients may lead to impaired cognitive function, which is also a common symptom among individuals with tinnitus.^44^ Tinnitus can also result in a decrease in serine protease. Serine protease is closely associated with mood and cognitive function, and a decline in its levels in the body may cause anxious behavior.^45^ Consequently, this could be a potential mechanism underlying the anxiety experienced by tinnitus patients.^46^ Another enzyme we examined is Aldo-Keto Reductase family 1 member C1 (AKR1C1), which primarily regulates the synthesis and clearance of sex hormones. Research has indicated that AKR1C1 can influence anxiety-related behaviors by modulating hormone levels in the body. Hence, tinnitus may cause an increase in AKR1C1 levels, potentially contributing to anxiety-related behaviors by affecting hormone levels in tinnitus patients.^47^

In this work, we have identified the enzymes responsible for promoting tinnitus and explored the enzymes associated with tinnitus complications. However, there are certain limitations that need to be addressed. Firstly, our focus was primarily on tinnitus patients in the GWAS database, which predominantly consists of individuals from the European population. This lack of diversity limits the representation of different populations. To validate our results, further research on non-Europeans is required.^48^ Additionally, the database did not provide detailed information on the duration of tinnitus in patients, making it difficult to determine the specific effects of the aforementioned proteases on tinnitus which last diverse durations. Despite the absence of a specified duration for tinnitus, this investigation serves as a valuable reminder of the potential therapeutic targets for tinnitus management. Regardless of whether the tinnitus is acute or chronic, there is an urgent requirement for experimental research to elucidate the precise therapeutic implications of enzymes across varying durations of tinnitus.^49^ Furthermore, tinnitus complications are predominantly observed in chronic tinnitus patients, necessitating further investigation into the impact of proteases on tinnitus. It is worth noting that the incidence of tinnitus is strongly influenced by gender,^50^ and therefore, our study was unable to differentiate the effect of gender on the association under investigation. Lastly, it is important to acknowledge that our findings are primarily based on statistical analysis, and thus, further validation is required to strengthen our conclusions.

## Conclusions

In this study, the causal relationship between 233 enzymes and tinnitus was investigated by MR method, and 5 kinds of proteases were found to have protective effect on tinnitus and 4 kinds of proteases to promote tinnitus. At the same time, the effects of tinnitus and some related enzymes in the body were also explored, and it was proved that tinnitus can reduce the content of 2 proteases and increase the content of 4 proteases in the body. These results lay a foundation for finding effective treatment targets for tinnitus, and also provide more options for the prevention and treatment of tinnitus-related complications.

## ORCID ID

Peng Liu: 0000-0002-2797-6949

Xinmiao Xue: 0000-0002-0005-4207

Zhixin Zhang: 0009-0002-2622-3301

Lijun Zhang: 0000-0002-1495-9832

Yu Peng: 0000-0001-5522-187X

Qi Song: 0000-0003-1331-3164

Fangyuan Wang: 0000-0002-9880-3930

## Funding

This work was supported by Medical Science Research Project of Hebei (20261409).

## Data availability statement

The authors declare that all data are available in repository.

## Declaration of competing interest

The authors declare no conflicts of interest.
